# Enolase 1 stimulates glycolysis to promote chemoresistance in gastric cancer

**DOI:** 10.18632/oncotarget.17868

**Published:** 2017-05-15

**Authors:** Xiaoling Qian, Wenxia Xu, Jinye Xu, Qiqi Shi, Jiaqiu Li, Yu Weng, Zhinong Jiang, Lifeng Feng, Xian Wang, Jianwei Zhou, Hongchuan Jin

**Affiliations:** ^1^ Laboratory of Cancer Biology, Key Lab of Biotherapy in Zhejiang, Sir Run Run Shaw Hospital, Medical School of Zhejiang University, Zhejiang, China; ^2^ Department of Medical Oncology, Sir Run Run Shaw Hospital, Medical School of Zhejiang University, Zhejiang, China; ^3^ Department of Clinical Medicine, Sir Run Run Shaw Hospital, Medical School of Zhejiang University, Zhejiang, China; ^4^ Department of Pathology, Sir Run Run Shaw Hospital, Medical School of Zhejiang University, Zhejiang, China; ^5^ Department of Molecular Cell Biology and Toxicology, School of Public Health, Nanjing Medical University, Nanjing, China

**Keywords:** enolase 1, cisplatin-resistance, glycolysis, gastric cancer, miRNA-22

## Abstract

Chemotherapy is the major choice for the cancer treatment of early and advanced stages. However, intrinsic or acquired drug resistance significantly restricts the clinical efficacy of chemotherapy. It is critical to develop novel approaches to detect and overcome drug resistance. In this study, we demonstrated that accelerated glycolysis played a pivotal role in both intrinsic and acquired cisplatin-resistance of gastric cancer cells. The metabolic reprogramming of cisplatin-resistant cells was characterized by increased glycolysis dependence. Inhibition of glycolysis with glucose starvation or 2-Deoxy-D-glucose (2-DG) treatment significantly reversed drug resistance. By proteomic screening, we found the increased expression of the glycolytic enzyme Enolase 1 (ENO1) in cisplatin-resistant gastric cancer cells. Depletion of ENO1 by siRNA significantly reduced glycolysis and reversed drug resistance. Moreover, the increased expression of ENO1 was attributed to the down-regulation of ENO1-targeting miR-22, rather than activated gene transcriptional or prolonged protein stability. Finally, the elevated levels of ENO1 proteins were associated with the shorter overall survival of gastric cancer patients. In conclusion, ENO1 is a novel biomarker to predict drug resistance and overall prognosis in gastric cancer. Targeting ENO1 by chemical inhibitors or up-regulating miR-22 could be valuable to overcome drug resistance.

## INTRODUCTION

Gastric cancer is one of the most prevalent cancers worldwide, especially in East Asia [1]. Chemotherapy in the form of neo-adjuvant or adjuvant therapy is the dominant treatment for most of the gastric cancers. Despite of the rapid development of new therapeutic drugs such as target therapy drugs, cisplatin (DDP) remains one of the most important drugs used in the treatment of gastric cancer. Unfortunately, drug resistance poses a major challenge to benefit gastric cancer patients from DDP-based chemotherapy.

Multiple mechanisms participate in DDP resistance, including increased drug efflux, drug inactivation, enhanced DNA damage repair, active survival signaling pathway and evasion of apoptosis [2]. Recently, the relevance of metabolic reprogramming to drug resistance received much attention [3, 4]. As one of major hallmarks of cancer cells, accelerated aerobic glycolysis contributes to DDP resistance in various cancers such as cervical cancer and lung cancer [5–11]. Although aerobic glycolysis produces less ATP per molecule of glucose than oxidative phosphorylation (OXPHOS), it can confer many selective advantages. For example, the ATP generation rate of glycolysis is almost 100 times faster than that of OXPHOS. By consuming more glucose, sufficient ATP could be produced rapidly from glycolysis [12]. In addition, intermediate products during glycolysis can be used as materials for the biosynthesis of macromolecules indispensable for cancer proliferation and growth, like nicotinamide adenine dinucleotide phosphate (NADPH), lipids and nucleic acids. Moreover, NADPH is instrumental to maintain sufficient levels of reduced forms of glutathione (GSH) to antagonize oxidative stress produced by DDP. Meanwhile, glycolysis can also enable cancer cells to reduce ROS generation by limiting the pyruvate flux into mitochondrial respiration, and thus acquire resistance to apoptosis or even promote metastasis [13–15].

To explore the mechanism of drug resistance in gastric cancers, we employed BGC823/DDP with acquired resistance and MGC803 intrinsically resistant to cisplatin [16]. We showed that increased glucose uptake and enhanced aerobic glycolysis occurred in gastric cancer cells with intrinsic or acquired resistance to DDP. Inhibition of glycolysis suppressed cell proliferation and reversed drug resistance. The enhanced glycolysis in drug resistance was caused by increased ENO1 expression resulted from the downregulation of miR-22. Overexpression of ENO1 or down-regulation of miR-22 enhanced glycolysis and promoted cisplatin-resistance. Meanwhile, depletion of ENO1 or up-regulation of miR-22 repressed glycolysis and restored cisplatin sensitivity. Therefore, targeting ENO1 or up-regulating miR-22 could be valuable to overcome drug resistance.

## RESULTS

### Glycolysis was enhanced in cisplatin resistant gastric cancer cells

**Figure 1:**
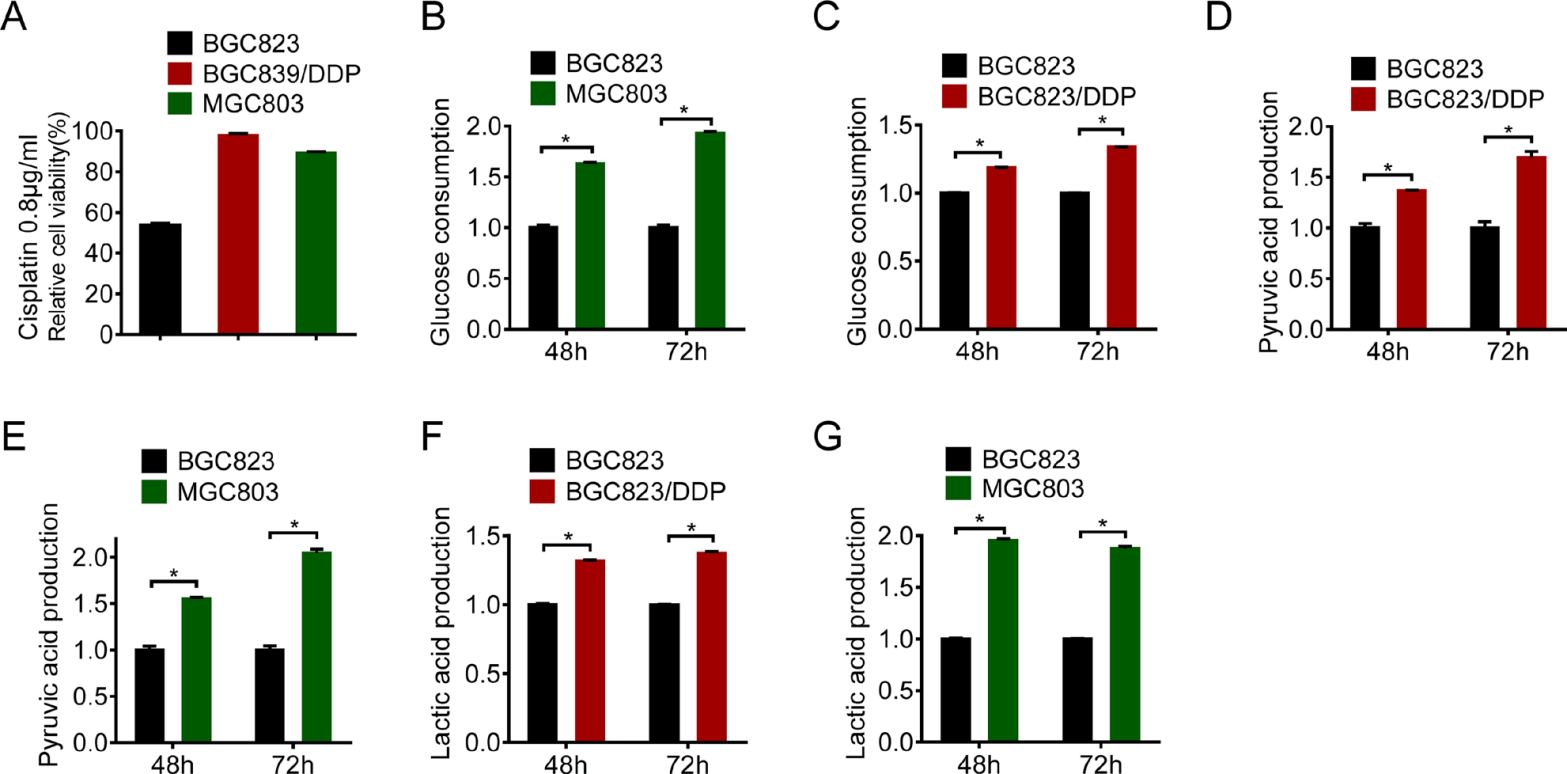
Glycolysis was enhanced in cisplatin resistant gastric cancer cells (**A**) Cell viability was assessed by MTS assay through cisplatin treatment (0.8 μg/ml) of BGC823, BGC823/DDP and MGC803 cells for 24 h. (**B**–**C**) Glucose consumption was measured between BGC823 and BGC823/DDP, BGC823 and MGC803. The fold changes were normalized by BGC823 glucose consumption (μmol/10^6^ cells). Pyruvic acid production (**D**–**E**) and lactic acid production (**F**–**G**) were analyzed according to instructions. The fold changes were normalized by BGC823 acid production (μmol/10^6^ cells). Results are from representative experiments in triplicate and shown as the mean ± S.D. *p < 0.05.

To explore molecular mechanisms responsible for drug resistance in gastric cancer, we employed BGC823/DDP with acquired resistance and MGC803 intrinsically resistant to cisplatin [16]. As shown in Figure 1A, there were more survived BGC823/DDP and MGC803 cells when compared with BGC823 cells upon treatment with 0.8 μg/ml of cisplatin. Since nutrient or energy metabolism has been reported to contribute to drug resistance [17–19], we compared glucose consumption in these cells. Both BGC823/DDP and MGC803 cells consumed more glucose than BGC823 cells (Figure 1B and 1C). Consistent with increased glucose consumption, there were more glycolysis metabolic products such as pyruvic acid and lactic acid produced in BGC823/DDP and MGC803 cells than BGC823 cells (Figure 1D, 1E, 1F and 1G). Taken together, these data demonstrated that the glycolysis was enhanced in cisplatin resistant gastric cancer cells.

### Inhibition of glycolysis reversed cisplatin resistance

**Figure 2:**
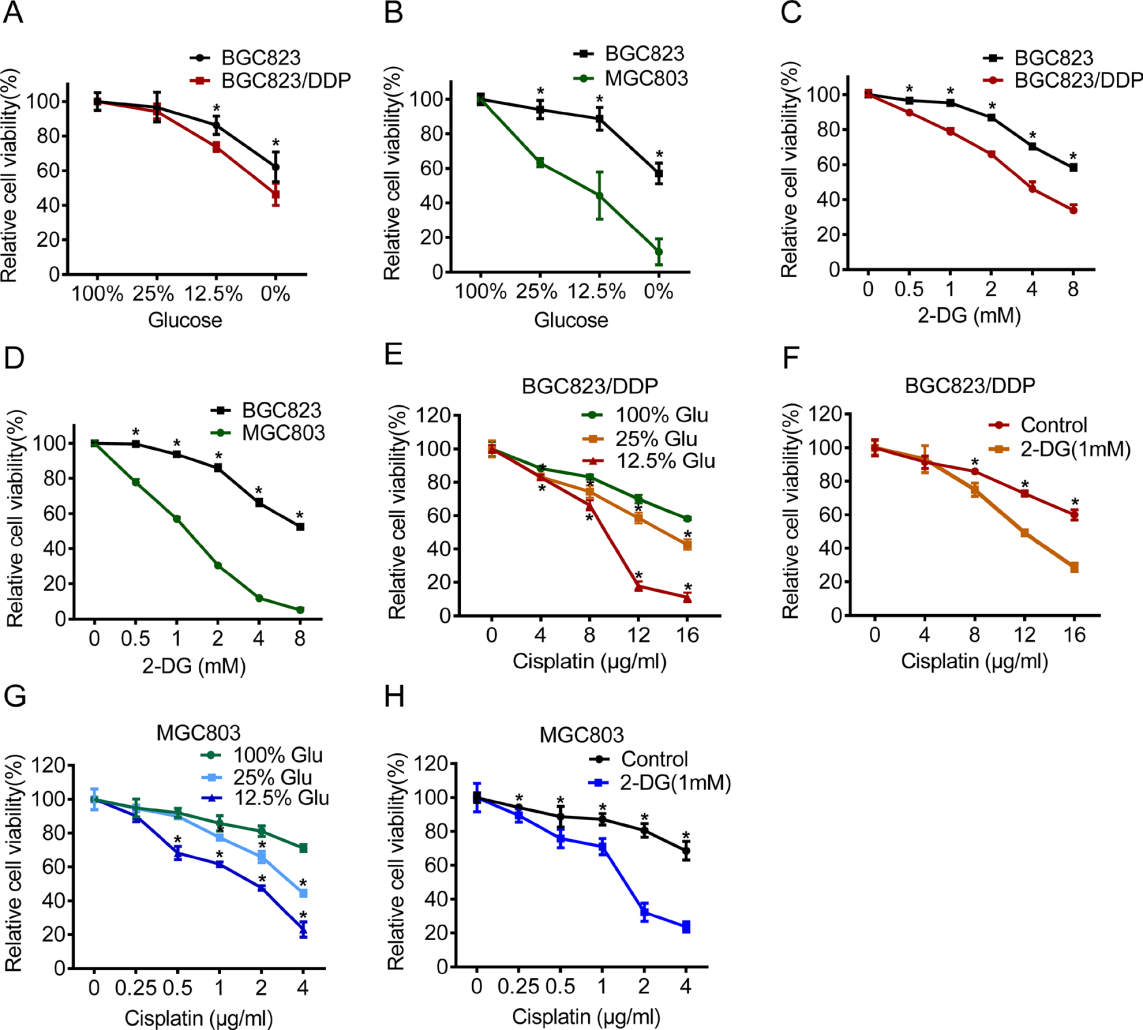
Inhibition of glycolysis reversed cisplatin resistance (**A**–**B**) BGC823, BGC823/DDP and MGC803 cells were cultured in different glucose concentrations of 0%, 12.5%, 25% and 100% for 48 h. Cell viability was assessed by MTS assay. (**C**–**D**) 2-DG was added at concentrations of 0 mM, 0.5 mM, 1 mM, 2 mM, 4 mM and 8 mM for 48 h and the cell viability was measured by MTS. (**E**) Glucose was added with concentrations of 12.5%, 25% or 100% for 48 h and during the last 24 h BGC823/DDP cells were exposed to 0 μg/ml, 4 μg/ml, 8 μg/ml, 12 μg/ml and 16 μg/ml cisplatin. The cell viability was measured by MTS. (**F**) BGC823/DDP cells were cultured in 1 mM 2-DG for 48 h and added by 0 μg/ml, 4 μg/ml, 8 μg/ml, 12 μg/ml and 16 μg/ml cisplatin for the last 24 h. The cell viability was measured by MTS. (**G**) Glucose was supplemented with concentrations of 12.5%, 25% and 100% for 48 h and during the last 24 h MGC803 cells were exposed to 0 μg/ml, 0.25 μg/ml, 0.5 μg/ml, 1μg/ml, 2 μg/ml and 4 μg/ml cisplatin, finally, cell survival was determined by MTS. (**H**) MGC803 cells were cultured in 1 mM 2-DG for 48 h and added by 0 μg/ml, 0.25 μg/ml, 0.5 μg/ml, 1 μg/ml, 2 μg/ml and 4 μg/ml cisplatin for the last 24 h. The cell viability was measured by MTS. Results are from representative experiments in triplicate and shown as the mean ± S.D. *p < 0.05.

Glycolysis provided metabolic products and energy for cell survival. To clarify the relevance of enhanced glycolysis to drug resistance, we applied glucose deprivation or 2-Deoxy-D-glucose (2-DG), the analogue of glucose as a competitive glycolytic inhibitor. Firstly, we found that BGC823/DDP and MGC803 cells were more sensitive to glucose deprivation than BGC823 cells (Figure 2A and 2B). Similarly, they were more sensitive to 2-DG treatment (Figure 2C and 2D). These results indicated that chemo-resistant cells were dependent more on glycolysis for survival.

**Figure 3:**
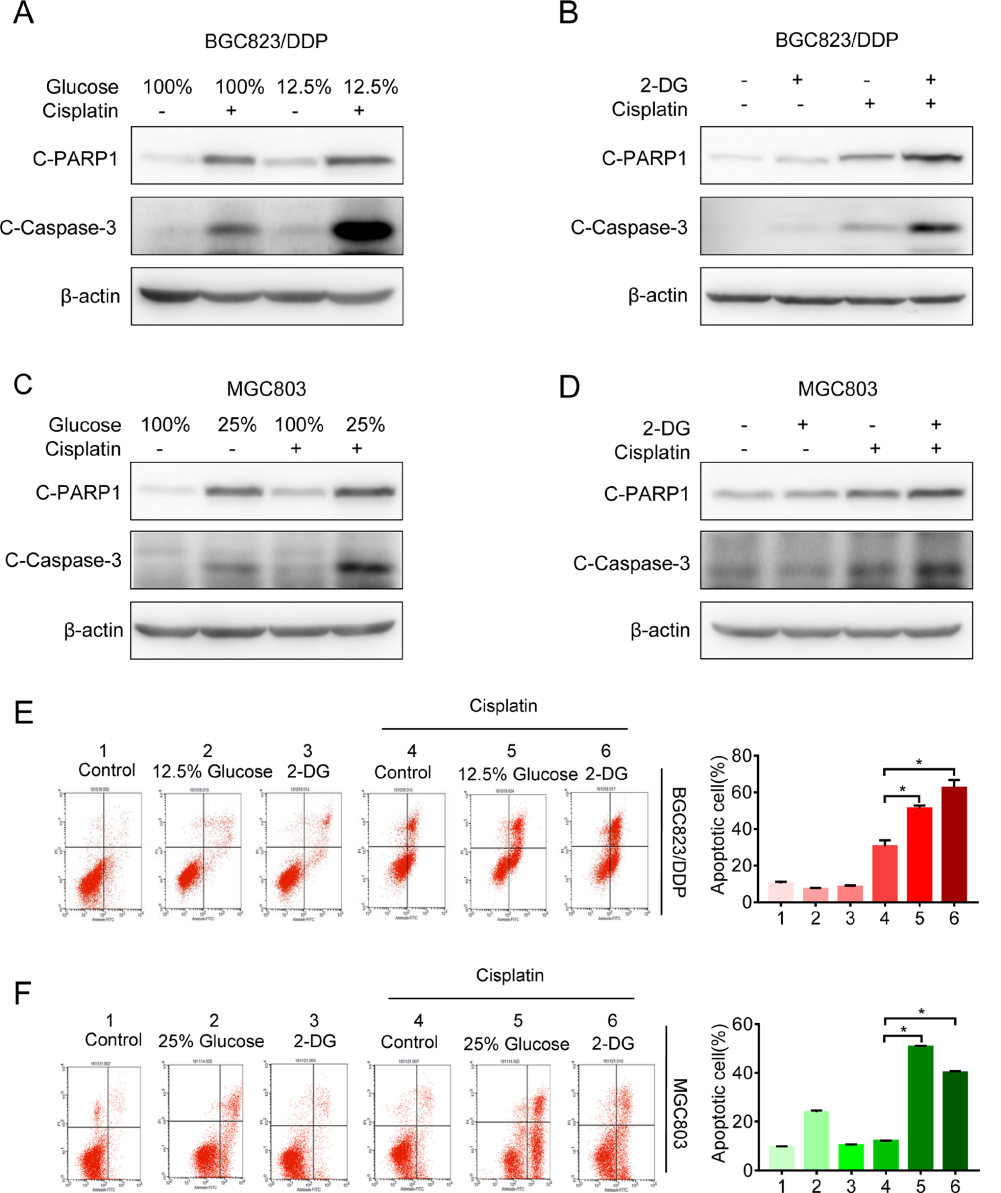
Inhibition of glycolysis reversed cisplatin resistance (**A**) The BGC823/DDP cells were exposed to 12.5% and 100% of glucose for 48 h with 8 μg/ml cisplatin exposure for the last 24 h. C-PARP1 and c-Caspase-3 were detected by Western blotting. β-actin served as loading control. (**B**) BGC823/DDP cells were treated with 2.5 mM 2-DG for 48 h and 8 μg/ml cisplatin for 24 h. C-PARP1 and c-Caspase-3 were determined by Western blotting. (**C**) MGC803 cells were exposed to 25% and 100% of glucose for 48 h with 2.5 μg/ml cisplatin for 24 h and determined by Western blotting. (**D**) MGC803 cells were treated with 1 mM 2-DG for 48 h and 2.5 μg/ml cisplatin for 24 h, then analyzed by Western blotting. (**E**) BGC823/DDP cells were treated with 2.5 mM 2-DG or 12.5% of glucose for 60 h, during the last 36 h, 8 μg/ml DDP was added to the media. Cell apoptosis was analyzed by flow cytometry analysis. (**F**) MGC823 cells were treated with 1 mM 2-DG and 25% of glucose for 60 h, during the last 36h, 2.5 μg/ml DDP was added to the media. Cell apoptosis was determined by flow cytometry analysis. Results are from representative experiments in triplicate and shown as the mean ± S.D. *p < 0.05.

Next, we investigated the effect of glycolysis inhibition on cisplatin resistance. We found that glucose deprivation markedly reversed cisplatin resistance in both BGC823/DDP and MGC803 cells (Figure 2E and 2G). Furthermore, 2-DG treatment also increased sensitivity to cisplatin in BGC823/DDP and MGC803 cells (Figure 2F and 2H). Both cleaved caspase-3 and cleaved PARP1 proteins were increased in glucose deprived or 2-DG-treated BGC823/DDP cells after cisplatin use (Figure 3A and 3B). Similarly, glucose deprivation or 2-DG treatment enhanced cisplatin-induced cleavage of caspase-3 and PARP1 in MGC803 cells (Figure 3C and 3D). The Annexin V/PI detection showed that apoptotic cells were apparently increased induced by cisplatin in glucose deprived or 2-DG treatment of BGC823/DDP and MGC803 cells (Figure 3E and 3F). In summary, inhibition of enhanced glycolysis could reverse cisplatin resistance.

### Cisplatin-resistant cells up-regulated ENO1 to enhance glycolysis

**Figure 4:**
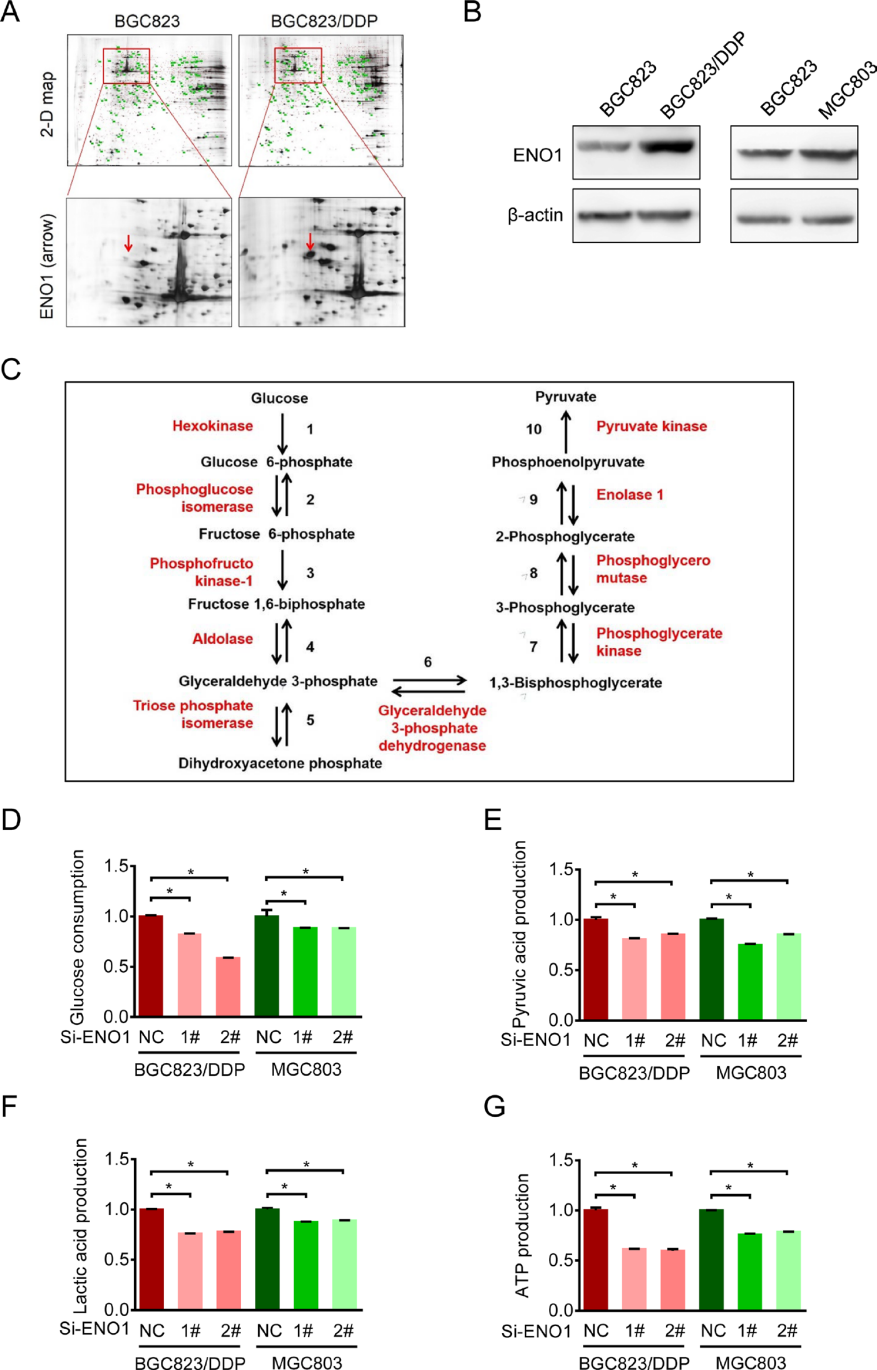
Cisplatin-resistant cells up-regulated ENO1 to enhance glycolysis (**A**) The expressions of ENO1 were assessed by 2-DE-MS in BGC823 and BGC823/DDP cells. The red arrows showed the location of ENO1. (**B**) ENO1 expressions in BGC823, BGC823/DDP and MGC803 cells were confirmed by Western blotting analysis. (**C**) The schematic flow chart showed 10 steps of glycolysis, and ENO1 catalyzed 2-phosphoglycerate (2-PG) to phosphoenolpyruvate (PEP). Enzymes are indicated by red font, two-way arrow means reversible reaction while one-way means irreversible. (**D**–**G**) BGC823/DDP and MGC803 cells were transiently transfected with con siRNA and ENO1 siRNA. After 36 h, the culture media were replaced by fresh media. After another 36 h, the supernatant were collected and the levels of glucose, pyruvic acid and lactic acid were measured according to the cell numbers, and another set of cell samples were lysed to estimate the levels of intracellular ATP. Results are from representative experiments in triplicate and shown as the mean ± S.D. *p < 0.05.

To identify potential mechanisms underlying enhanced glycolysis and chemoresistance, we employed two-dimensional electrophoresis (2-DE) combined with MALDI-TOF MS to discover the proteomic differences between BGC823 and BGC823/DDP cells [16]. Among 40 identified differential spots, ENO1 was detected in BGC823/DDP cells but not in BGC823 cells (Figure 4A). Western blotting analysis further confirmed that ENO1 was significantly overexpressed in BGC823/DDP and MGC803 cells compared with BGC823 cells (Figure 4B). Since ENO1 was a key enzyme in glycolysis to catalyze 2-phosphoglycerate to phosphoenolpyruvate (Figure 4C), we wondered whether it was relevant to enhanced glycolysis and chemoresistance.

First, we compared changes in glycolysis in BGC823/DDP and MGC803 cells before and after ENO1 knockdown. After transient silencing of ENO1, the glucose consumption was decreased, especially in BGC823/DDP cells (Figure 4D). Consistently, the production of pyruvic acid, lactic acid and ATP were significantly decreased after ENO1 knockdown (Figure 4E, 4F and 4G). In conclusion, the increased ENO1 expression enhanced glycolysis in drug-resistant gastric cancer cells.

### ENO1 knockdown increased sensitivity to cisplatin

**Figure 5:**
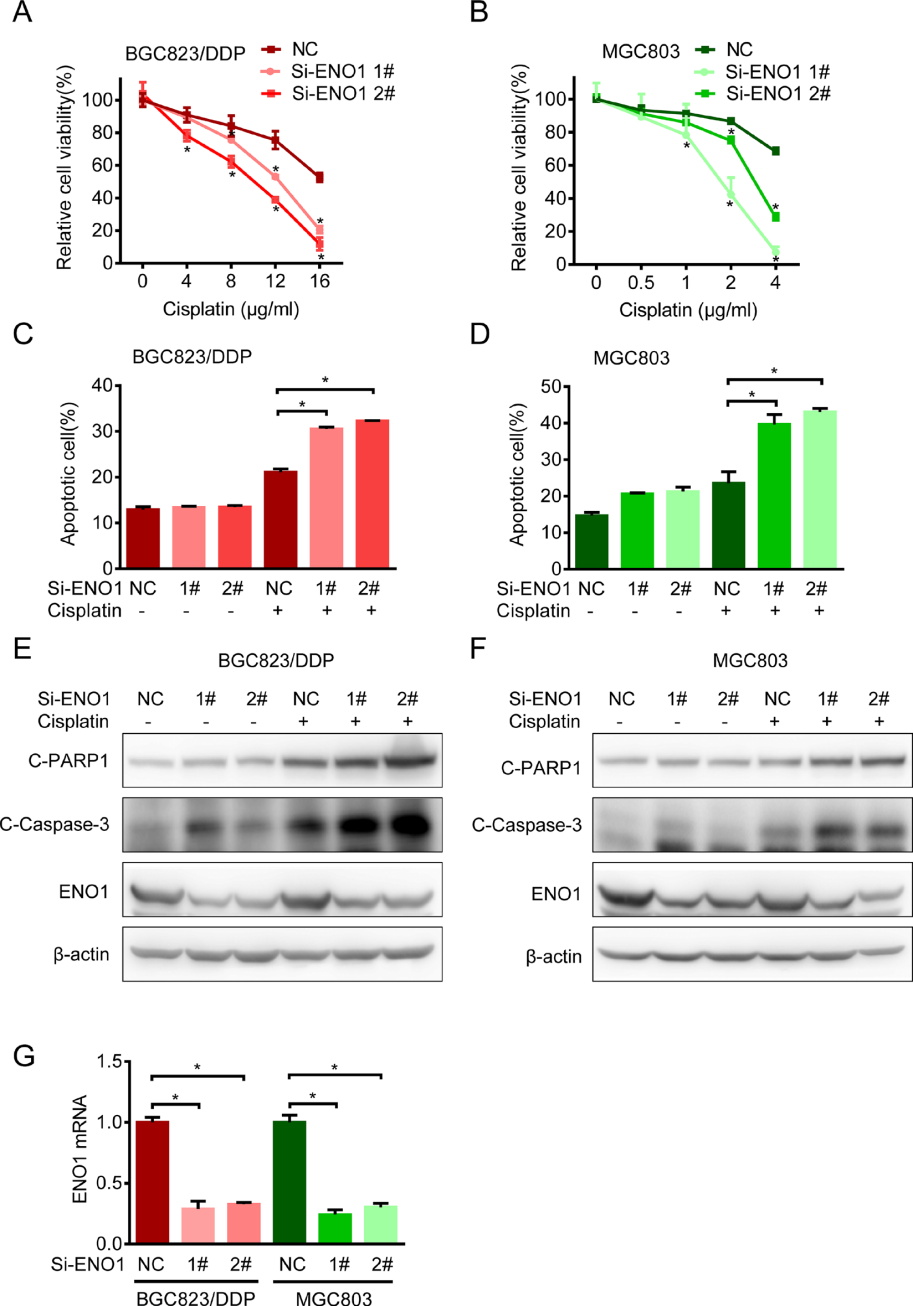
Knockdown ENO1 increased sensitivity to cisplatin (**A**) BGC823/DDP cells were transfected with siRNA-ENO1 for 48 h and treated with different concentrations of cisplatin for another 24 h. The cell viability was determined by MTS. (**B**) MGC803 cells were transfected with siRNA-ENO1 for 48 h and treated with different concentrations of cisplatin for another 24 h. The cell viability was determined by MTS. (**C**–**D**) The apoptosis of BGC823/DDP and MGC803 transfected with siRNA-ENO1 in combination with cisplatin (8 μg/ml DDP for BGC823/DDP and 2.5 μg/ml DDP for MGC803) for 36 h were estimated with flow cytometry analysis. (**E**–**F**) BGC823/DDP and MGC803 cells were transfected with siRNA-ENO1 for 48 h following cisplatin exposure for 24 h (8 μg/ml DDP for BGC823/DDP and 2.5 μg/ml DDP for MGC803). C-PARP1, c-Caspase-3 and ENO1 were determined by Western blotting. (**G**) The knockdown efficiency was shown by QPCR after 72 h transfection. Results are from representative experiments in triplicate and shown as the mean ± S.D. *p < 0.05.

Then we investigated the impact of ENO1 on cisplatin resistance. Upon ENO1 knockdown, cisplatin sensitivities in BGC823/DDP and MGC803 cells were significantly increased (Figure 5A and 5B). Such increases in drug sensitivities were accompanied with the activation of cell apoptosis, as evidenced by Annexin V and PI staining (Figure 5C and 5D) as well as increased cleavage of caspase-3 and PARP1 by Western blot (Figure 5E and 5F). The ENO1 knockdown efficiency was shown by QPCR (Figure 5G). In summary, these data suggested that the increased expression of key glycolysis catalytic enzyme ENO1 contributed to cisplatin resistance.

### Overexpression of ENO1 in cisplatin-sensitive cells induced cisplatin-resistance by glycolysis promotion

**Figure 6:**
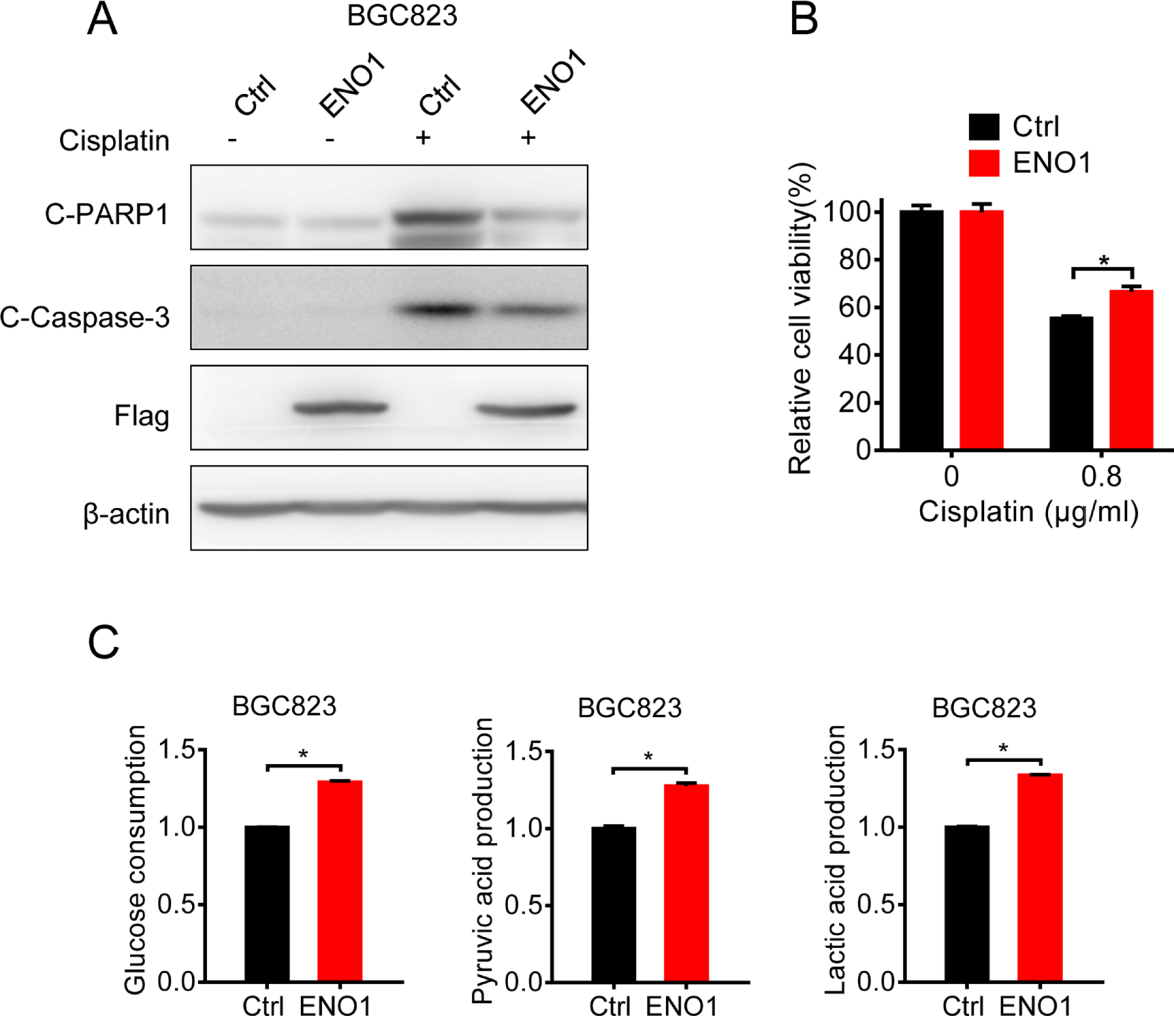
Overexpression of ENO1 in cisplatin-sensitive cells induced cisplatin-resistance by glycolysis promotion (**A**) BGC823 cells were transfected with Flag-ENO1 plasmids for 48 h following 0.8 μg/ml cisplatin exposure for 24 h. C-PARP1, c-Caspase-3, Flag-ENO1 and β-actin were assessed by Western blotting. (**B**) BGC823 cells were transfected with Flag-ENO1 for 48 h and treated with 0.8 μg/ml cisplatin for another 24 h. The cell viability was measured by MTS. (**C**) BGC823 cells were transiently transfected with ENO1 plasmids. After 36 h, the culture media were replaced by fresh media. After another 36 h, the supernatant were collected and the levels of glucose, pyruvic acid and lactic acid were measured according to the cell numbers. Results are from representative experiments in triplicate and shown as the mean ± S.D. *p < 0.05.

Then we explored the influence of ENO1 on cisplatin-sensitive gastric cells. Once ENO1 was overexpressed in BGC823 cells, the cleavage of caspase-3 and PARP1 were decreased and less viability inhibition occurred after cisplatin treatment (Figure 6A and 6B). Consistently, the consumption of glucose and the production of pyruvic acid and lactic acid were significantly increased (Figure 6C), indicating that ENO1 induced cisplatin-resistance by promoting glycolysis.

### microRNA-22 targeted ENO1 mRNA

**Figure 7:**
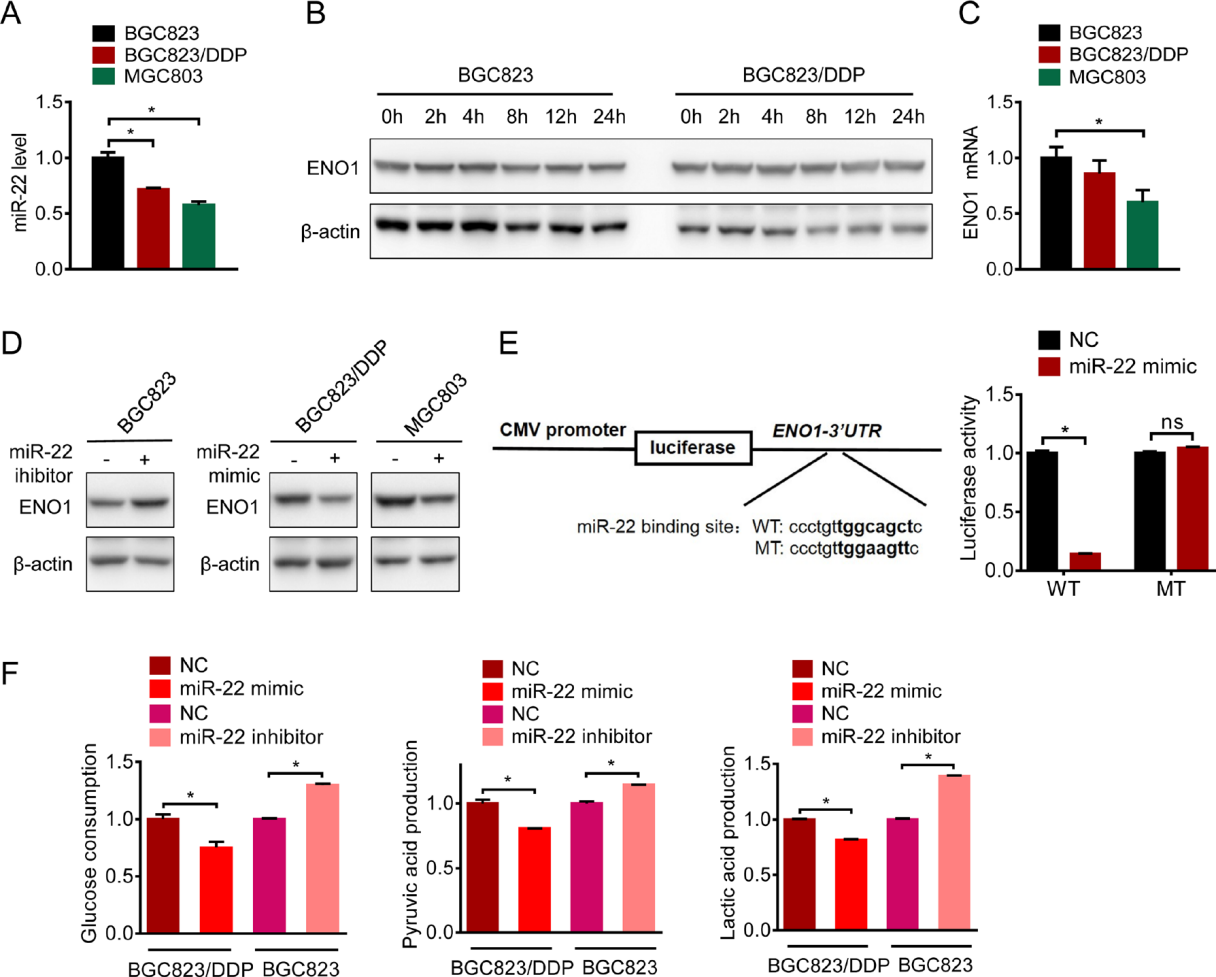
microRNA-22 targeted ENO1 mRNA (**A**) ENO1 mRNA of BGC823, BGC823/DDP and MGC803 cells were evaluated by QPCR. (**B**) BGC823 and BGC823/DDP cells were treated with 50 μg/ml cycloheximide (CHX) to block new protein synthesis for 0 h, 2 h, 4 h, 8 h, 12 h and 24 h. ENO1 was determined by Western blotting. (**C**) QPCR was used to detect miRNA-22 in untreated BGC823, BGC823/DDP and MGC803 cells. (**D**) miRNA-22 mimic and inhibitor were transfected into certain cells for 72 h, and ENO1 protein were tested by western blot. (**E**) The wild type and the mutant luciferase plasmids, all of which contained 3′-UTR segment of ENO1 were transfected into BGC823/DDP cells combined with miR-22 mimic for 48 h, and luciferase activities were tested. (**F**) BGC823 cells and BGC823/DDP cells were transiently transfected with miR-22 inhibitor or mimic respectively. After 36 h, the culture media were replaced by fresh media. After another 36 h, the supernatant were collected and the levels of glucose, pyruvic acid and lactic acid were measured according to the cell numbers. Results are from representative experiments in triplicate and shown as the mean ± S.D. *p < 0.05; ns means no statistical significance (*p* > 0.05).

To clarify the regulation of ENO1 in cisplatin-resistant cells, we determined the mRNA level of ENO1. Real-time quantitative RT-PCR showed slightly decreased level of mRNA in resistant cells (Figure 7A). In addition, there were no changes in the protein half-lives of ENO1 in BGC823 and BGC823/DDP cells (Figure 7B), indicating that the increased expression of ENO1 in drug resistance was not attributed to the enhanced transcription of ENO1 gene or prolonged stability of ENO1 protein. Therefore, we hypothesized that microRNAs (miRNA) might be involved in the regulation of ENO1. Based on on-line prediction (www.microRNA.org), we chose the highly-conserved microRNA with the highest mirSVR score, miR-22, for further investigations. Indeed, miR-22 was down-regulated in both resistant cells (Figure 7C). Moreover, miR-22 mimics reduced ENO1 protein expression in BGC823/DDP and MGC803 cells (Figure 7D), while miR-22 inhibitor significantly up-regulated ENO1 in BGC823 cells (Figure 7D). The 3′-UTR of the ENO1 mRNA with a complementary binding site for miR-22 was cloned into the luciferase vector (Figure 7E, left panel). And then we constructed a mutant plasmid which contained point mutations within the miR-22 binding site. Importantly, miR-22 mimics markedly repressed the luciferase reporter activity driven by the wild-type ENO1 3’-UTR while the luciferase expression of mutant plasmid was not affected by miR-22 mimics (Figure 7E, right panel), indicating that ENO1 was a *bona fide* target of miR-22. Then we analyzed the influence of miR-22 on glycolysis. The consumption of glucose as well as the production of pyruvic acid and lactic acid were increased in BGC823 cells after transfection of miR-22 inhibitor (Figure 7F). In contrast, consumption of glucose and the production of pyruvic acid and lactic acid were reduced by miR-22 mimic in BGC823/DDP cells. Taken together, these results suggested that miR-22 was responsible for the overexpression of ENO1 protein and glycolysis enhancement in drug-resistant gastric cancer cells.

### ENO1 predicted a poor clinical outcome in gastric cancer

**Figure 8:**
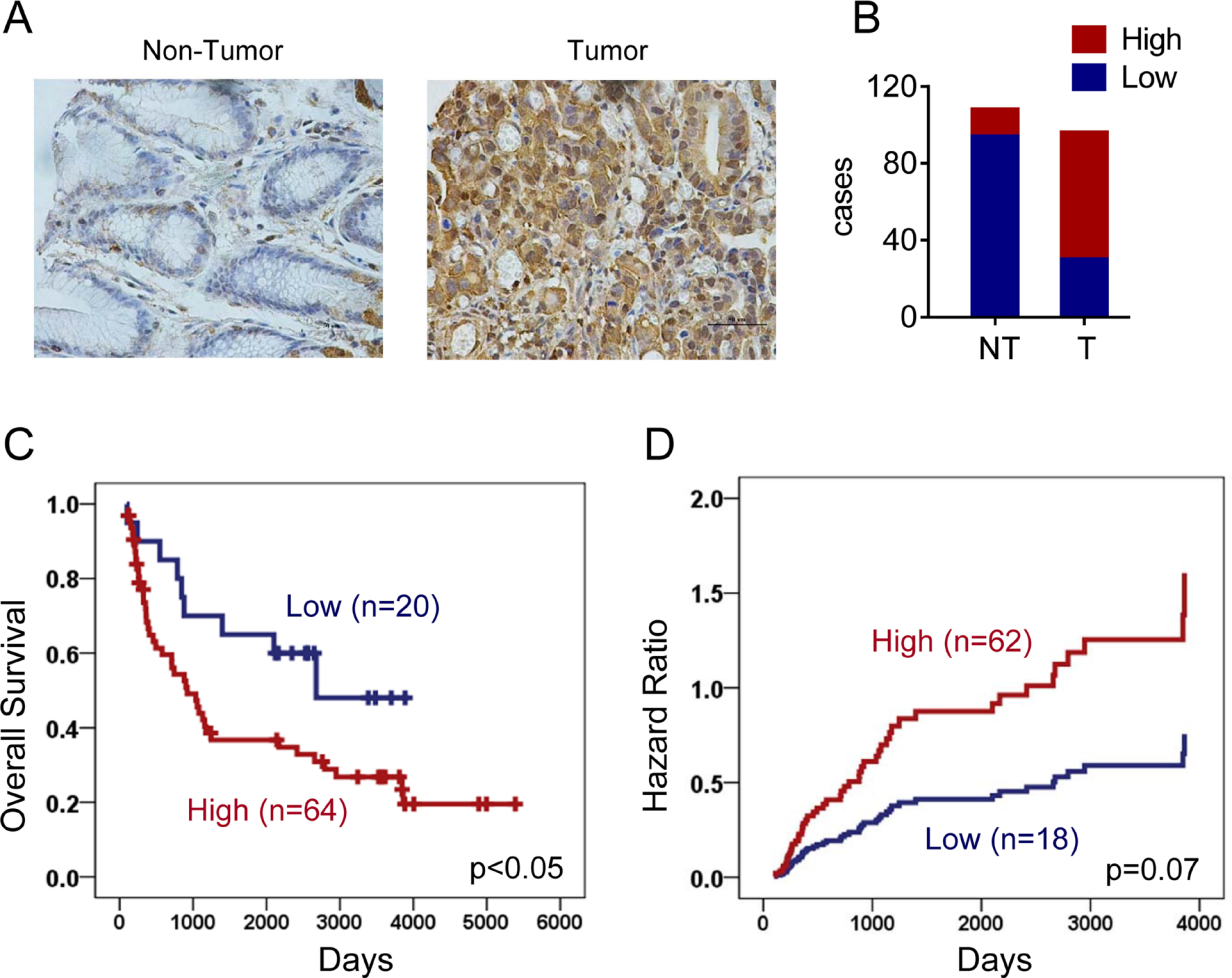
ENO1 predicted a poor clinical outcome in gastric cancer (**A**) Expressions of ENO1 in normal and tumor gastric tissues were determined by immunohistochemistry staining. (**B**) Statistical results showed patients of gastric cancer expressed high level of ENO1. (**C**) Overexpression of ENO1 protein predicted shorter overall survival (OS) of gastric cancer patients (*p* < 0.05). (**D**) Multivariate Cox regression indicated high ENO1 expression associated with shorter survival.

We next explored the clinical relevance of ENO1 in gastric cancer patients. ENO1 expression in primary gastric adenocarcinoma and non-tumor tissues were evaluated by immunohistochemistry staining. As shown in Figure 8A and 8B, ENO1 expression was increased in tumor tissues (high expression rate: 13% in non-tumor tissues and 69% in tumor tissues). The association of ENO1 expression levels with various clinicopathologic characteristics in gastric cancer patients was summarized in Table 1. Interestingly, ENO1 protein was highly expressed in male patients (high expression rate: 85% in male and 58% in female, Chi-square test, *p* < 0.05). But its expression levels were not significantly associated with TNM stage and differentiation (Chi-square test, *p* > 0.05). Moreover, high expression of ENO1 had a strong association with shorter overall survival (OS) (Low ENO1 expression: 2532 days, 95% CI: 1861–3203; High ENO1 expression: 1931 days, 95% CI: 1406–2457, Log-Rank test, *p* < 0.05, Figure 8C). Univariate Cox regression analysis revealed that high ENO1 expression significantly increased two folds of death hazard (*p* < 0.05, Table 2). When other potential prognosis factors such as TNM stage, differentiation and gender were incorporated in the multivariate Cox regression, high ENO1 expression also displayed the trend to indicate shorter survival (*p*=0.07, Figure 8D and Table 2). In contrast, ENO1 expression had no influence on progression free survival (PFS) (Low ENO1 expression: 788 days, 95% CI: 520–1056; High ENO1 expression: 780 days, 95% CI: 318–1242, Log-Rank test, *p* > 0.05 and Table 3).

**Table 1:** ENO1 expression in gastric cancer

Variable	Low expression	High expression	*p*-value
**Gender**			0.045
F	17	37	
M	8	45	
**TNM**			0.109
I	7	11	
II	9	23	
III	5	35	
IV	2	11	
**Differentiation**			0.877
Low	15	50	
Moderate or High	10	31	

**Table 2:** Cox regression analysis of overall survival in gastric cancer

Variable	Univariate		Multivariate	
	RR (95% CI)	*p*-value	RR (95% CI)	*p*-value
Gender				
Female	1.00	0.946	1.00	0.986
Male	1.0 (0.6–1.6)		1.0 (0.6–1.8)	
Differentiation				
Moderate or High	1.00	0.322	1.00	0.657
Low	1.3 (0.8–2.1)		1.2 (0.6–2.2)	
TNM Stage				
I	1.00	< 0.01	1.00	< 0.01
II	5.7 (2.0–16.5)		4.8 (1.4–17.0)	
III	5.4 (2.0–15.2)		5.0 (1.4–17.2)	
IV	10.4 (3.3–32.2)		10.4 (2.7–39.4)	
ENO1 expression				
Low	1.00	< 0.05	1.00	0.07
High	2.1 (1.0–4.3)		2.1 (0.9–2.2)	

**Table 3:** Cox regression analysis of progression free survival in gastric cancer

Variable	Univariate		Multivariate	
	RR (95% CI)	*p*-value	RR (95% CI)	*p*-value
Gender				
Female	1.00	0.557	1.00	0.452
Male	0.9 (0.5–1.4)		0.8 (0.4–1.5)	
Differentiation				
Moderate or High	1.00	0.105	1.00	0.320
Low	1.5 (0.9–2.6)		1.4 (0.7–2.9)	
TNM Stage				
I	1.00	< 0.01	1.00	< 0.01
II	4.7 (1.8–12.4)		3.5 (1.1–11.5)	
III	5.2 (2.0–13.5)		4.4 (1.4–13.7)	
IV	12.8 (4.4–37.1)		9.8 (2.9–33.4)	
ENO1 expression				
Low	1.00	0.884	1.00	0.912
High	0.9 (0.5–1.9)		1.1 (0.5–2.3)	

## DISCUSSION

As one of hallmarks of human cancer cells, elevated uptake of glucose and accelerated glycolytic rates were first observed by Germany Biochemist Dr. Otto Heinrich Warburg and later confirmed by many studies [7, 20, 21].

Anti-cancer treatments like radiation and chemotherapy can induce free radicals to kill cancer cells. Therefore, enhanced glycolysis may contribute to drug resistance by increasing reducing capacity [8–10, 19, 22]. In addition, the intracellular microenvironment affected by elevated glycolysis can turn to be lower glucose concentration with high levels of lactate, pyruvate and ATP, thus facilitating the development of multiple drug resistance (MDR) [23–25]. The low glucose concentration in microenvironment increases the expression of glucose transporters and activates PI3K/AKT/mTOR to inhibit apoptosis and facilitate cellular survival [3, 26]. Elevated lactate levels can reinforce DNA repair and promote cisplatin resistance in cervical carcinoma cells via the inactivation of histone deacetylase [27]. In addition, pyruvate can promote chemoresistance by up-regulating the expression of p-glycoprotein to enhance the efflux of chemotherapeutic drugs [28]. ATP was considered as a pivotal determinant in maintaining MDR phenotype partially because elevated ATP levels directly activated transporters with ATP-binding cassettes to exclude intracellular cytotoxic drugs [29]. As a result, depletion of ATP could eventually reverse chemoresistance [4, 8].

In this study, we found enhanced glycolysis in both intrinsic and acquired cisplatin-resistant gastric cancer cells. Both glucose consumption and lactate production were increased in gastric cancer cells resistant to the well-used chemotherapeutic drug. Inhibition of glycolysis not only inhibited cell proliferation but also reversed resistance to cisplatin. Therefore, gastric cancer cells with drug resistance also developed dependence on enhanced glycolysis for cellular survival. There are many mechanisms proposed to contribute to the Warburg effect. For example, aberrant activation of oncogenic signaling pathways including Ras/MAPK, PI3K/AKT/mTOR or loss of function of cancer suppressor genes like p53 which could promote the expression of glucose transporters and glycolytic enzymes [30–32]. Meanwhile, the metabolic products such as lactate can in turn promote glycolysis as a positive feedback [33]. Here, we identified another rate-limiting enzyme overexpression in both acquired and intrinsic drug-resistant gastric cancer cells by proteomic screening. ENO1 protein, which catalyzes 2-phosphoglycerate to phosphoenolpyruvate, was significantly overexpressed in BGC823/DDP and MGC803 cells.

In mammals, enolase has three isoforms, that is alpha-enolase, beta-enolase and gamma-enolase, encoded by three distinct genes [34]. Alpha-enolase (ENO1) is an essential glycolytic enzyme detected in nearly all adult human tissues. It has been reported that ENO1 was up-regulated in various human cancers including gastric cancer [35, 36], glioma [37], breast cancer [38], lung cancer [39], head and neck cancer [40], endometrial carcinoma [41], pancreatic adenocarcinoma [42, 43], and Non-Hodgkin's Lymphomas (NHLs) [44]. Our results further confirmed these findings. ENO1 protein was highly expressed in tumor tissues in comparison with adjacent non-tumor tissues including gastric tissues with dysplasia (Figure 8 and data not shown). Interestingly, we also found that ENO1 expression was further increased in both acquired and intrinsic drug resistance. Depletion of ENO1 inhibited glycolysis and reversed drug resistance.

So far, glycolytic enzymes also have been reported in promoting drug resistance by other mechanisms in addition of activating metabolic flux [45–48]. For instance, hexokinase (HK), one of the rate-limiting enzymes in glycolysis, can locate to mitochondrial outer membrane and inhibit mitochondria-dependent apoptosis [49, 50]. Similarly, ENO1 can also locate in nucleus to inhibit the transcription of c-myc, a proto-oncogene, which acts as a negative transcription factor [51]. However, we failed to detect the enhanced nuclear location of ENO1 in either BGC823/DDP or MGC803 cells (date not shown). Therefore, the stimulation of drug resistance by increased ENO1 expression in gastric cancer was most likely attributed to its enzyme activity to regulate glycolysis rather than its function as a transcription regulator. Inhibition of glycolysis by ENO1 depletion reduced glucose consumption, and the production of lactate, pyruvate and ATP, thus reversing drug resistance. How inhibition of glycolysis reversed drug resistance remains to be clarified. However, it has been reported that inhibition of ENO1 could induce autophagy to arrest cellular growth [52]. In addition, many metabolites including pyruvate were able to reprogram gene expression in an epigenetic manner [53]. Therefore, the understanding of differential gene expression before and after ENO1 depletion by high throughput methods would be valuable to understand the regulation of drug resistance by glycolysis.

Studies have revealed that high level of ENO1 was strongly linked to poor prognosis of cancer patients [35, 37, 38, 44]. Consistently, we indeed found that high ENO1 expression predicted shorter overall survival of gastric cancers. Therefore, ENO1 was proposed to be a potential tumor biomarker of chemoresistance and overall prognosis [39, 41, 43]. Certainly, its clinical value warrants further validations with a larger cohort.

The increased expression of ENO1 in some cancers was resulted from high mRNA levels. Actually, ENO1 is a direct transcriptional target of HIF-1α [19, 54, 55]. However, to our surprise, mRNA of ENO1 were even slightly decreased in MGC803 and BGC823/DDP cells. Interestingly, high level of ENO1 protein with low ENO1 mRNA level was also detected in methotrexate-resistant breast cancer cells [56]. We indeed found that the discrepancy between protein and mRNA of ENO1 was attributed to the downregulation of ENO1-tagreting miR-22, but not the post-translational regulation of its protein stability. Interestingly, miR-22 played different roles in tumorigenesis depending on caner types [57–60]. However, miR-22 seemed to serve as a novel tumor suppressor in gastric cancers. MiR-22 expression was significantly reduced in gastric cancer tissues when compared with normal adjacent mucosa, and the reduced expression of miR-22 indicated shorter overall survival for patients [61]. Overexpression of miR-22 could inhibit gastric cancer growth [62–64]. These data were consistent with our findings, and we further discovered that low expression of miR-22 promoted glycolysis and cisplatin-resistance by up-regulating ENO1. It remains unclear how miR-22 expression was reduced in gastric cancers. Interestingly, STAT5 could down-regulate miR-22 expression by binding the promoter of its host gene. Inhibition of Jak3, STAT3 and STAT5 triggered overexpression of miR-22 and suppressed cancer proliferation in cutaneous T-Cell lymphoma [65]. Therefore, miR-22 could be valuable to target ENO1 to overcome drug resistance in gastric cancers.

In conclusion, gastric cancers could develop a cisplatin-resistant phenotype by glycolysis acceleration and dependence. Glucose deprivation or glycolysis inhibition can sensitize cells to chemotherapy. ENO1 serves as a master regulator of tumor glycolysis and predicts an unfavorable prognosis for gastric cancers. MiR-22 targets 3′-UTR of ENO1 to repress its expression and reduced expression of miR-22 eventually promote ENO1 expression to enhance glycolysis and drug resistance. Therefore, inhibition of ENO1 or up-regulation of miR-22 could restrain glycolysis to increase cisplatin sensitivity.

## MATERIALS AND METHODS

### Cells, antibodies, chemicals, culture medium and plasmids

Human gastric cancer cell lines BGC823 and MGC803 were obtained from the Type Culture Collection of the Chinese Academy of Sciences (Shanghai, China). The culture media used were RPMI 1640 containing 10% of fetal bovine serum, 100 U/ml of penicillin and 100 μg/ml of streptomycin (Invitrogen, Carsbad, CA, USA). The cells were grown in a humidified incubator (37°C, 5% CO_2_). The conditional glucose media were mixed by RPMI 1640 complete medium (11 mM glucose) and RPMI 1640 glucose-free medium (Gibico, Grand Island, NY, USA) according to proportions of 0%, 12.5%, 25% and 100% at a certain ratio, containing dialyzed fetal bovine serum (Gibico, Grand Island, NY, USA, NZ origin). Cisplatin was obtained from Sigma-Aldrich (St. Louis, MO, USA) and stored at room temperature in darkness. The cisplatin-resistant BGC823/DDP cells were derived from the parental BGC823 cells as previously described [16]. In short, BGC823 cells were persistently exposed to increasing concentration gradient of cisplatin from 0.05 μg/ml to 1 μg/ml. Before each experiment, BGC823/DDP cells were grown in cisplatin-free RPMI 1640 media for 2 weeks. Antibody for ENO1 was bought from Proteintech (Rosemont, PA, USA). Antibodies for β-actin, cleaved Caspase-3 and cleaved PARP1 were from Cell Signaling Technology (Boston, MA, USA). Antibody for Flag was from Sigma (St.Louis, MO, USA). 2-DG, CHX, MTS and other chemicals were all bought from Sigma-Aldrich (St. Louis, MO, USA). The human ENO1 ORF mammalian expression plasmid was constructed on the vector pCMV3-N-FLAG and purchased from Sino Biological Inc (Beijing, China, Catalog Number: HG11554-NF).

The pMIR-Luciferase-REPORT-ER vectors were obtained from Applied Biosystems (Foster City, CA, USA).

### SiRNA, miRNA mimics/inhibitors and plasmids transfection

The ENO1 siRNAs were synthesized by Genepharma (Shanghai, China) for transient knockdown and listed in Table 4. Briefly, cells were seeded in 6-well plates overnight, and subsequently transfected with siRNA or miRNA mimics/inhibitors using lipofectamine RNAiMax (Invitrogen, Carsbad, CA, USA) and the plasmid DNA with X-GENE (Roche Diagnostics, Indianapolis, IN, USA) according to the manufacturer's instructions. The media were changed after 24 h transfection and the total time for transfection was 72 h.

**Table 4:** Sequences of oligos used in the study

Name	Sequence
h-ENO1-RT-PCR	F:TGAGGGAATGAGTGACGGC
	R:ACAGCCTTTGAGACACCCTTC
h-β-Actin-RT-PCR	F:GCTATCCCTGTACGCCTCTG
	R:AGGAAGGAAGGCTGGAAGAG
hsa-miR-22-3p	AAGCTGCCAGTTGAAGAACTGT
ENO1-3′ UTR-F	GGAGAGCTCGCTGTGGGCAGGCAAGC
ENO1-3′ UTR-R	GCGAAGCTTCTCATGGGTCACTGAGGCTTTTT
ENO1-3′ UTR-mutant-F	CTCCCTGGAGCCCTGTTGGAAGTTCTAGCTTTGCA
ENO1-3′ UTR-mutant-R	TGCAAAGCTAGAACTTCCAACAGGGCTCCAGGGAG
ENO1 siRNA 1#	5′-GGAGAAAUAUGGGAAAGAUTT-3′
ENO1 siRNA 2#	5′-CCCAGUGGUGUCUAUCGAATT-3′
hsa-miR-22-3p mimic	AAGCUGCCAGUUGAAGAACUGU
hsa-miR-22-3p inhibitor	ACAGUUCUUCAACUGGCAGCUU

### RNA isolation, reverse transcription and quantitative PCR

Total RNA was extracted by Trizol reagent (Invitrogen, Carsbad, CA, USA) and miRNA was extracted by MIRNeasy Mini Kit (QIAGEN Gmbh, Hilden, Germany) following the manufacturer's instructions. RNA concentrations were measured by absorbance (A260) on NanoDrop 1000 (Nanodrop, Wilmington, DE, USA). 1 μg of total RNA was performed reverse transcription reaction with High Capacity cDNA Reverse Transcription kit (Applied Biosystems, Foster City, CA, USA, 4375222). The quantitative realtime PCR was conducted by using SYBR Green Master Mix (Applied Biosystems, Foster City, CA, USA) to determine mRNA levels while miScript PCR system (QIAGEN, Hilden, Germany) for microRNA expression analysis respectively. β-actin and U6 were used for the normalization of mRNA and microRNA respectively. The primers used in this study were listed in Table 4.

### Two-dimensional electrophoresis and mass spectrometry

2-DE and mass spectrometry (MS) were performed as previously described [16]. Briefly, same amount of samples of BGC823 cells and BGC823/DDP cells were loaded on the 2-D gels, and then gels were stained with silver. The following criteria were used to identify differentially expressed proteins: spot intensity ≥ 2-fold increase or decrease in BGC823/DDP cells in comparison with BGC823 cells. The proteins were analyzed by Swiss-Prot and NCBI non-redundant databases.

### Luciferase activity assay

3′-UTR segment of the ENO1 with wild or mutant miR-22 binding sites were amplified by polymerase chain reaction (PCR) and inserted into the pGEM-T Easy vector (Promega Corporation, Madison, WI, USA) for sequence validation. The correct insert was again cloned into the pMIR-Luciferase-REPORTER vectors. The restriction enzymes were Sac I and Hind III. The primers used were shown in Table 4. The resultant plasmids were co-transfected with miR-22 mimics by using lipofectamine RNAiMax. After 48 h transfection, the luciferase activities were measured by the Dual-GLO Luciferase Assay System (Promega Corporation, Madison, WI, USA).

### Cell viability assay

Cells viability was determined by Cell Titer 96^®^AQueous Cell Proliferation Assay kit (MTS assay) (Promega Corporation, Madison, WI, USA). 0.8 × 10^4^ BGC823 cells and BGC823/DDP cells or 0.6 × 10^4^ MGC803 cells were seeded per well in the 96-well plates. After 24 h, cells were subjected to different concentrations of 2-DG (0.5 mM, 1 mM, 2 mM, 4 mM and 8 mM) or glucose (0%, 12.5%, 25% and 100%) for 48 h with or without DDP. The conditioned glucose media were mixed by RPMI 1640 complete medium (11 mM glucose) and RPMI 1640 glucose-free medium according to proportion of 0%, 12.5%, 25% and 100%. BGC823/DDP was treated with 4 μg/ml, 8 μg/ml, 12 μg/ml and 16 μg/ml DDP while MGC803 with 0.5 μg/ml, 1 μg/ml, 2 μg/ml and 4 μg/ml DDP respectively for 24 h.

### Flow cytometry analysis

3 × 10^5^ BGC823/DDP and 2 × 10^5^ MGC803 cells were seeded in 6-well plates, and the next day changed for 2-DG (2.5 mM for BGC823/DDP and 1 mM for MGC803) or hypoglycemic media (12.5% glucose for BGC823/DDP and 25% glucose for MGC803) for 60 h, until the last 36 h, BGC823/DDP cells were added with 8 μg/ml DDP and MGC803 with 2.5 μg/ml DDP respectively. Apoptotic cell death was determined by using the FITC Annexin V Apoptosis Detection Kit I (BD Bioscience, Bedford, MA, USA) according to the manufacturer's instruction. Cells were washed twice with cold PBS and then suspended at a concentration of 1 × 10^6^ cells/ml in 1 × Binding Buffer. Then 100 μl cellular suspensions were added with 5 μl of FITC Annexin V and 5 μl PI, and then incubated for 15 min at room temperature in darkness before analyzed by flow cytometry.

### Western blotting

Western blot analyses were performed as previously described [66]. Equal amounts of proteins were separated by SDS-PAGE and transferred to PVDF membrane. Interested proteins were probed with the indicated primary antibodies followed by incubation in HRP-conjugated corresponding secondary antibodies and revealed by enhanced chemiluminescence (Millipore, Billerica, MA, USA).

### Glucose consumption

Cells were plated in the six-well plates, after 24 h the culture media were replaced by 3 ml fresh media with different treatments. Then after certain period, the supernatant were collected and glucose concentrations were measured by Abbott ArchitectC16000 (Abbott Park, North Chicago, Illinois, USA). The cells left were trypsinized and counted for three times. The glucose consumption levels were standardized to μmol/10^6^ cells. The fold changes were normalized by BGC823 consumption.

### Lactic acid and pyruvic acid measurement

The supernatant of cells were collected after treatment and lactic acid and pyruvic acid were measured by colorimetric method following the manufacturer's instructions of Lactic Acid assay kit and Pyruvate assay kit (Nanjing Jiancheng bioengneering institute, Nanjing, China). The cell numbers were counted for three times, and finally, the acid production were the measured by μmol/10^6^ cells.

### Intracellular ATP measurement

Briefly, cells were seeded for 24 h and then replaced by fresh media with different treatments. After cultivation, media were removed, cells lysates were measured using luciferase-based ATP Assay Kit (Beyotime, Shanghai, China) by following the assay instructions for intracellular ATP levels. The unit of measurement was nmol/10^6^ cells.

### Immunohistochemical staining

The paraffin sections were prepared from gastric tissue biochips, containing normal, precancerosis and tumors. The indirect streptavidinperoxidase method was used for detecting protein ENO1 expression level according to manufacturer's introduction. The antibody was rabbit anti-ENO1 antibody (1:1000, Proteintech, Rosemont, PA, USA). The stained specimens were evaluated separately by two pathologists. The scores were based on expression intensity and its proportion, and finally divided into four groups, none (0), weak (1), medium (2) and strong (3). Score 0-1 was defined as low expression while Score 2–3 as high.

### Statistical analysis

All values were expressed as the mean ± S.D. The statistical significance of the differences between groups was determined by the parametric unpaired Student's *t-test*. Statistical significance was accepted if *p* < 0.05.

## Abbreviations

ATP: adenosine triphosphate
caspase-3: cysteine-aspartic acid protease-3
CHX: cycloheximide
DDP: cis-diammine dichloride-platinum(II)
ENO1: Enolase 1
GSH: glutathione
Glu: Glucose
MDR: multiple drug resistance
miRNA: microRNA
MS: mass spectrometry
MT: mutant
NADPH: nicotinamide adenine dinucleotide phosphate
OS: overall survival
OXPHOS: oxidative phosphorylation
PARP1: poly(ADP-ribose) polymerase 1
PCR: polymerase chain reaction
PFS: progression free survival
PI: phosphatidylinositol
ROS: reactive oxygen species
RT-PCR: reverse transcription polymerase chain reaction
2-DE: two-dimensional electrophoresis
2-DG: 2-Deoxy-D-glucose.
